# Coffee berry borer (Coleoptera: Scolytidae) population dynamics across Hawaii Island’s diverse coffee-growing landscape: optimizing location-specific pesticide applications

**DOI:** 10.1093/jee/toae061

**Published:** 2024-04-05

**Authors:** Melissa A Johnson, Nicholas C Manoukis

**Affiliations:** Daniel K. Inouye US Pacific Basin Agricultural Research Center, United States Department of Agriculture–Agricultural Research Service, 64 Nowelo St., Hilo, HI 96720, USA; Daniel K. Inouye US Pacific Basin Agricultural Research Center, United States Department of Agriculture–Agricultural Research Service, 64 Nowelo St., Hilo, HI 96720, USA

**Keywords:** elevation gradient, infestation, integrated pest management, phenology, trap catch

## Abstract

A major challenge to area-wide management of coffee berry borer (*Hypothenemus hampei* Ferrari) (Coleoptera: Scolytidae) is understanding how a heterogeneous coffee-growing landscape affects coffee berry borer population dynamics across temporal and spatial scales. We examined coffee phenology, weather, coffee berry borer flight activity, infestation, coffee berry borer position within the fruit, and management across 14 commercial coffee farms from 2016 to 2018 on Hawaii Island to characterize variation among districts and elevations. Here we aim to determine whether the timing of pesticide applications might be optimized based on specific locations. We observed larger populations of coffee berry borer at low-elevation farms and in the Kona district compared to mid- and high-elevation farms and the Ka’u district. Temperature, relative humidity, and rainfall all differed significantly across districts and elevations. We also observed a trend of higher fruit production at low-elevation farms compared to high-elevation farms, and differences in the timing of fruit development. Infestation increased with higher pest pressure and air temperatures and reduced fruit availability early and late in the season. Lastly, the timing and number of management interventions varied among districts and elevations. Combining information on trap catch, infestation, coffee berry borer position, and plant phenology, we present an optimized pesticide spray schedule for each location and find that the number of sprays could be reduced by 33–75% in comparison to the existing integrated pest management recommendations while maintaining effective control. Implementing a coordinated area-wide approach refined by small-scale optimization will lead to improved management of coffee berry borer on individual farms and a reduction in pest pressure across the coffee-growing landscape.

## Introduction

**Fig. 1.**
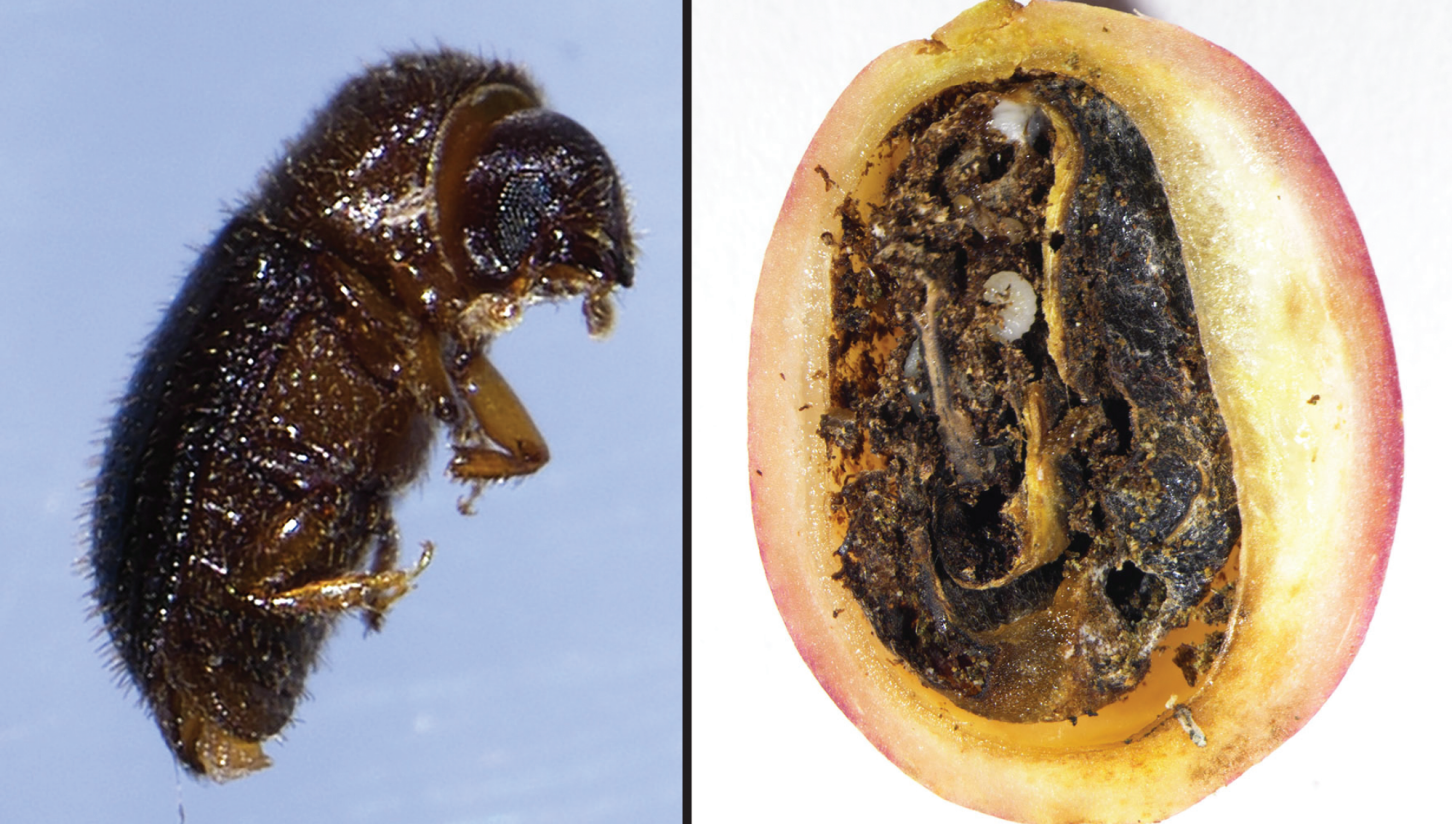
Adult female coffee berry borer approximately 2 mm in length (left), and infested coffee fruit showing bean damage from coffee berry borer larval feeding (right).

Native to Africa, coffee berry borer (*Hypothenemus hampei* Ferrari) (Coleoptera: Scolytidae) is considered the world’s most damaging insect pest of coffee, causing more than $500 million in annual losses globally (Vega et al. 2002). The adult female coffee berry borer bores a hole into the coffee fruit (“berry”) and eventually into the seed (“bean”) where it excavates tunnels for reproduction (Damon 2000). The larvae feed on the endosperm tissue within the seed as they develop (Fig. 1), resulting in reduced yields and quality (Le Pelley 1968, Duque and Baker 2003, Vega et al. 2015). Male and female coffee berry borer siblings mate in their natal berry, and then mated females leave in search of a new berry to infest (Baker et al. 1992, Mathieu et al. 1997). Chemical control must be conducted during the narrow time frame when the females are exposed and initiating boring, as this is when they are most vulnerable to pesticide sprays (Damon 2000). If left unmanaged, coffee berry borer populations can rapidly multiply and infest more than 90% of the coffee crop (Johnson and Manoukis 2020).

Historically, very little management was required to produce a profitable coffee crop in Hawaii, with the main agronomic activities including pruning, weed management, fertilization, and harvesting (Kinro 2003, Bittenbender and Smith 2008). The establishment of coffee berry borer in 2010 drastically changed the way coffee was grown and managed in the islands (Lee et al. 2023). In addition to the usual activities needed to maintain the farm, growers today often must monitor regularly for coffee berry borer, apply multiple sprays of the biopesticide *Beauveria bassiana*, conduct frequent and efficient harvesting, and implement postharvest sanitation to prevent the complete destruction of the crop (Aristizábal et al. 2016, 2017a, 2023a, Johnson et al. 2020, Kawabata et al. 2020). The nearly continuous mosaic of coffee (including feral plants, abandoned, unmanaged, and managed farms) across the Kona and Ka’u districts of Hawaii Island allow coffee berry borer population reservoirs to persist year-round, with poorly managed farms often serving as sources of coffee berry borer that reinfest neighboring farms (Johnson and Manoukis 2020). With more than 800 coffee farms on Hawaii Island alone, area-wide integrated pest management (IPM) strategies are needed to successfully reduce coffee berry borer population densities at the landscape level.

To develop successful area-wide IPM programs, it is critical to understand how coffee berry borer populations persist and disperse across the landscape (Rodríguez et al. 2013). Field studies conducted in Hawaii over the last 13 years have provided location-specific information on coffee berry borer development (Hamilton et al. 2019), flight activity (Messing 2012, Aristizábal et al. 2017b, Johnson and Manoukis 2021), infestation (Aristizábal et al. 2017b, Johnson and Manoukis 2020), postharvest reservoirs (Johnson et al. 2019), *Beauveria bassiana* efficacy and spray strategies (Greco et al. 2018, Hollingsworth et al. 2020, Woodill et al. 2021, Wraight et al. 2021, 2022), biological controls (Brill et al. 2021, Castrillo et al. 2020, Follett et al. 2016, 2023, Hollingsworth et al. 2011, Kawabata et al. 2016, Sim et al. 2016, Wraight et al. 2018, 2022, Yousuf et al. 2021), physical controls (Johnson et al. 2020), chemical controls (Kawabata et al. 2023), and cultural controls (Aristizábal et al. 2023b). Still, there remain gaps in our knowledge of coffee berry borer population dynamics over space and time, limiting our understanding of the relative importance of site-specific differences among farming districts and elevations in this small but economically important and world-renowned coffee-growing region.

In the present study, we characterized coffee phenology, coffee berry borer flight activity, fruit infestation, and coffee berry borer position within the fruit, weather, and management practices across a broad elevational gradient in the 2 major coffee-growing districts of Hawaii Island. We analyze these data to determine whether IPM recommendations should be updated based on location-specific differences in coffee berry borer population dynamics that are driven by weather, plant phenology, and management practices.

## Materials and Methods

### Study Sites

**Fig. 2.**
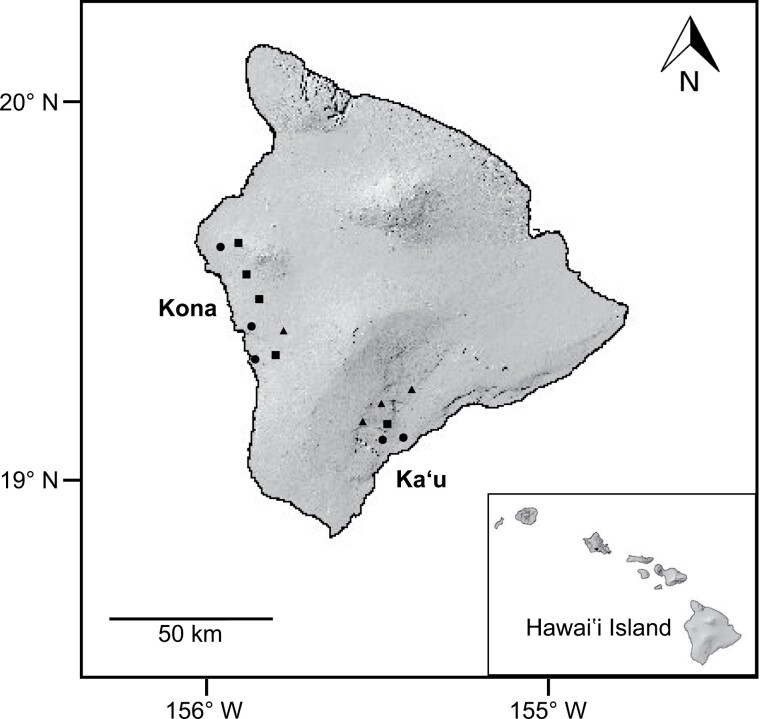
Map of Hawaii Island showing study sites in the Kona and Ka’u districts. Symbols refer to site elevation: circle = low (200–399 m), square = mid (400–599 m), and triangle = high (600–799 m). Inset depicts location of Hawaii Island within the Hawaiian archipelago.

Fourteen commercial coffee farms were selected for the study on Hawaii Island. Eight farms were located on the West side of the island in the Kona district, and 6 farms were on the Southeast side of the island in the Ka’u district (Fig. 2). While coffee is grown throughout the Hawaiian Islands, Kona and Ka’u are the 2 primary coffee-growing regions. Farms were selected to encompass the broad range of elevations and climatic conditions under which coffee is grown on Hawaii Island. In Kona, 8 farms ranged in elevation from 204 to 607 m, and in Ka’u, 6 farms ranged from 279 to 778 m in elevation. Farms between 200 and 399 m in elevation were defined as low elevation (*n* = 5), farms between 400 and 599 m were defined as mid-elevation (*n* = 5), and farms between 600 and 799 m were defined as high elevation (*n* = 4).

All farms were actively managed for coffee production throughout the study, although the timing and number of interventions varied. Management practices included regular pruning, weed management, fertilizer and pesticide application, cherry (ripe fruit) harvesting, and end-of-season strip-picking. Farms were largely characterized as sun-grown although some farms had scattered fruit, nut, or ornamental shade trees planted as well. All farms had *Coffea arabica* var. *typica* planted with the exception of one farm in Ka’u that had primarily var. *caturra* planted. Permission was obtained from each private landowner prior to initiating the study. Farm management records were collected regularly throughout the study period (March 2016–December 2018). Information was gathered on the timing and frequency of pruning, pesticide applications, and harvesting rounds.

### Coffee Berry Borer Flight Activity

To investigate coffee berry borer flight activity, red funnel traps (CIRAD, Montpellier, France) were randomly distributed within each site, with the number of traps used dependent on the size of the site (3–5 traps for farm plots 0.5–1.4 acres in size, 6–9 traps for farm plots 1.5–2.5 acres in size). Traps were hung on stakes and positioned ~1 m above the ground. Each trap was equipped with 40 ml of a 3:1 methanol:ethanol lure placed in semi-permeable plastic bags, and a collection cup filled with an aqueous kill solution of propylene glycol (see additional details in Johnson et al. 2018). Traps were checked biweekly throughout the year, and the contents collected in 70% ethanol. Lures were refilled as needed, and kill solutions were replaced biweekly. In the laboratory, coffee berry borer were separated from all other insects and counted under a stereomicroscope (Leica Microsystems GmbH, Wetzlar, Germany).

### Fruit Production and Phenology

From March to December, coffee plants were assessed biweekly for fruit production and phenology, coffee berry borer infestation, and coffee berry borer position. Tree sampling followed the methods developed by Bustillo et al. (1998) and modified by Johnson et al. (2018). Briefly, sampling grids were first established to ensure an even distribution of sampling of trees throughout each site. The number of trees sampled depended on the size of the study area (8–15 trees sampled at farm plots 0.5–1.4 acres, 18–25 trees sampled at farm plots 1.5–2.5 acres). To assess fruit production and phenology, a single branch was randomly selected at chest height from each tree and the number of infestable fruits (green and ripe fruits that were pea-size and larger) was counted.

### Infestation and Coffee Berry Borer Position

Coffee berry borer infestation was assessed by counting the total number of green berries per branch, then examining green fruits for an entrance hole in the central disc and recording the number of infested berries per branch. When present, 1–3 infested green fruits were collected from each branch and stored at 14 °C. Infested fruits were dissected within 24 h under a dissecting microscope at 30–50× (Leica, Microsystems GmbH, Wetzlar, Germany). We recorded the number of fruits in which the founding female was in the AB position (i.e., has commenced boring into the fruit but has not entered the endosperm) or CD position (i.e., has entered the endosperm and is building galleries for reproduction) (Bustillo et al. 1998). The AB position signals coffee berry borer flight and boring activity and is used to estimate when sprays will be most effective, while the CD position is used to estimate bean damage (Aristizábal et al. 2016, 2017a). For the coffee berry borer in the AB position, we also distinguished between females that were alive vs. dead to estimate the need for pesticide applications more accurately. Monitoring results from traps and infestation assessments were shared with growers after each survey, and it was at the farm owner’s discretion to spray or not based on this information.

### Weather Variables

Manual or cell-service weather stations were set up at each farm to measure air temperature, relative humidity (RH), and rainfall. Manual stations consisted of a Hobo Pro v2 temperature/RH data logger (U23-002, Onset Computer Corporation, Bourne, MA, USA) housed in a solar shield (RS3, Onset Computer Corporation, Bourne, MA, USA), and a rain gauge equipped with a manual data logger (RainLog 2.0, RainWise Inc.). Cell-service weather stations were comprised of a 4G remote monitoring station (RX3004-00-01, Onset Computer Corporation, Bourne, MA, USA) equipped with a temperature/RH sensor (S-THB-M002) and rain gauge (S-RGB-M002). Sensors were located between 1 and 3 m above the ground at each weather station, depending on the type of sensor.

### Spray Determination

A scoring matrix was developed to determine optimal times to control coffee berry borer using sprays of biopesticides such as *B. bassiana* or other commercially available chemical controls (e.g., pyrethrins, imidacloprid, spinosyns; see Kawabata et al. 2020 for a review of products approved for Hawaii) that target adults in the AB position (Supplementary information S1). For each district and elevation, scores were calculated from data collected on trap catch, fruit production, infestation, and AB position. Each of these 4 variables was given a score of 0–3 (0 = not a good time to spray, 3 = best time to spray) according to the range of values displayed in Table 1. The sum of these scores was tallied out of a total of 12 possible points for each biweekly sampling period (24 total points possible for each month). Each month was then assigned a spray priority ranking as follows: critical = 20–24 points (>80th percentile), high = 18–19 points (71th–80th percentile), medium = 16–17 points (60th–70th percentile), and low = 0–15 (<60th percentile).

**Table 1.** Scoring matrix used to determine optimal pesticide spray times to control coffee berry borer in Hawaii. Scores ranged from 0 (not a good time to spray) to 3 (best time to spray) for each variable. The 4 most important variables for determining spray times were as follows: trap catch (coffee berry borer/trap/day), fruit production (green berries/branch), fruit infestation (%), and coffee berry borer in the AB position (%), which indicates the early stages of boring when the coffee berry borer are exposed

Score	Trap catch	Fruit production	Coffee berry borer infestation	AB position
3	>30	>20	1–9	>30
2	20–30	10–20	10–19	20–30
1	10–19	1–9	20–30	10–19
0	0–9	<1	>30	0–9

### Data Analysis

All statistical analyses were conducted in R v. 4.2.3 (R Core Team 2018). Trap counts were converted to the mean number of coffee berry borer caught per trap per day and log-transformed (log + 1) prior to analysis. For each site, the daily maximum, mean, and minimum were estimated for air temperature and RH using the “aggregate” function in the *stats* package. We used the same method to estimate daily cumulative rainfall. We then used these daily values to calculate the mean air temperature (°C), mean RH (%), and cumulative rainfall (millimeter) for each 14-day sampling period.

For each variable, we then calculated the average across the 3 years sampled at each site. The assumption of normality for each variable was assessed using quantile–quantile plots and a Shapiro–Wilks test; an *F* test was conducted to check for equal variances using the *stats* package. Paired Wilcoxon signed-rank tests were used to compare each variable between districts, while Kruskal–Wallis tests were used to compare each variable across elevations. A Dunn’s test was then used for follow-on analyses of pairwise differences.

To characterize the influence of pest pressure (inferred through coffee berry borer trap catch), fruit availability, and weather on infestation, we built a generalized linear mixed model (GLMM) using the function *lme* in the *lme4* package v. 1.1-34 (Bates et al. 2015). The response variable for the GLMM was the proportion of infested berries (arc-sin transformed), with year and site (farm) as random effects. Fixed effects included the biweekly averages for coffee berry borer/trap/day (log-transformed), green berries per branch, temperature, RH, and cumulative rainfall. We assumed a Gaussian error distribution and an identity link function for the model. A backward reduction of fixed effects was done systematically, and log-likelihood values were compared to determine the best-fit model.

## Results

### Fruit Production and Phenology

**Fig. 3.**
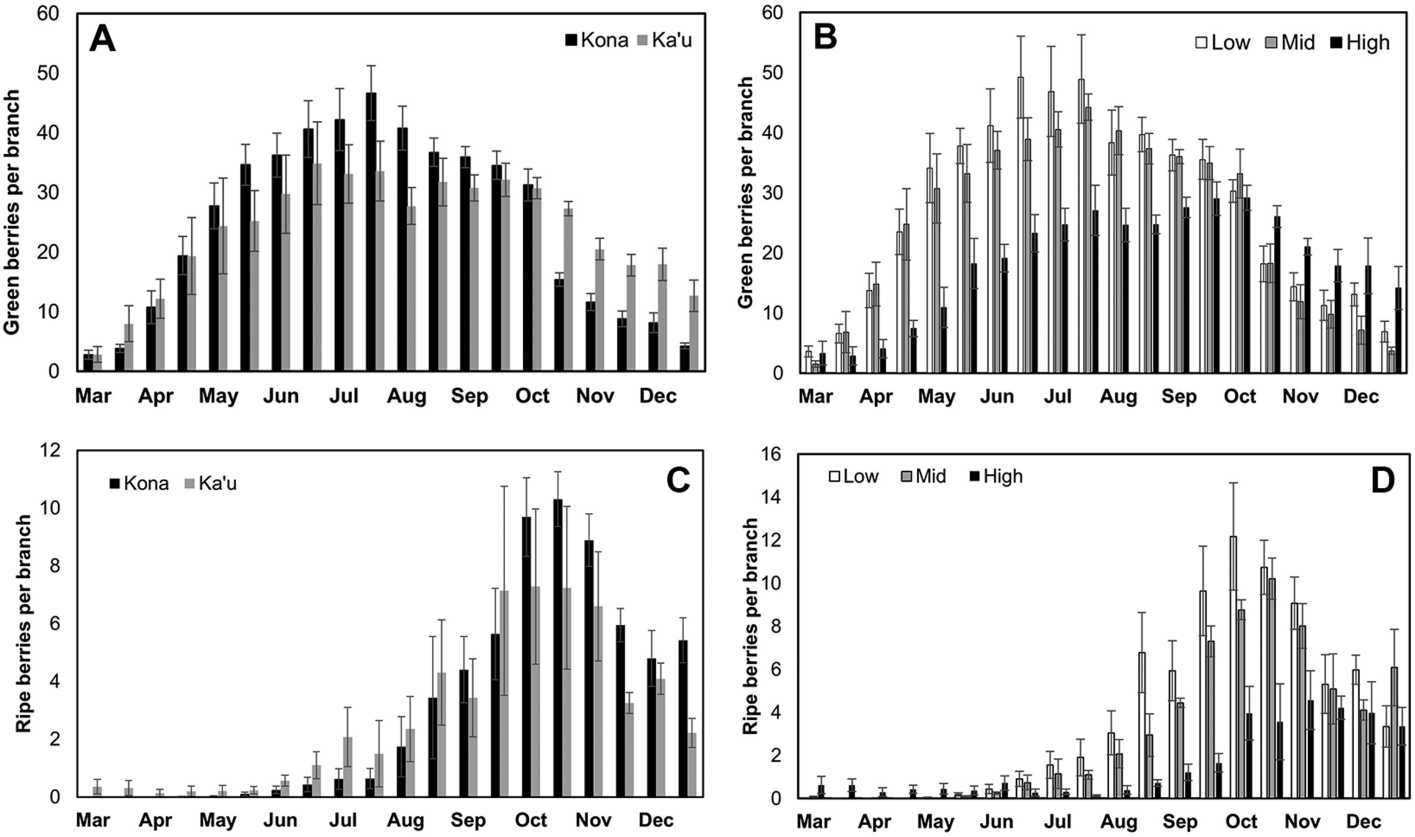
Mean coffee fruit production and phenology from 2016 to 2018 on Hawaii Island: A) green fruit in the Kona and Ka’u districts, B) green fruit at low, mid, and high elevations, C) ripe fruit in the Kona and Ka’u districts, and D) ripe fruit at low, mid, and high elevations. Error bars represent ± 1 standard error.

Fruit production at any given point in the season was not significantly different between Kona and Ka’u (25.66 vs. 24.18 green fruits/branch; *V* = 124, *P* = 0.50) (Fig. 3A). For both districts, fruit production peaked in June–July during the study years. Fruit production also did not differ significantly among elevations, although there was a trend of increased fruit production at low elevations compared to high elevations (low = 27.48, mid = 25.62, high = 18.65 green fruits/branch; χ^2^ = 4.69, df = 2, *P* = 0.09) (Fig. 3B). Fruit production peaked from June to July in low-elevation farms, June to August in mid-elevation farms, and September to October in high-elevation farms. Fruit ripening also did not differ between districts (Kona = 3.12 vs. Ka’u = 2.73 ripe fruits/branch; *V* = 113, *P* = 0.78) or among elevations (low = 3.86, mid = 3.12, high = 1.59; χ^2^ = 1.02, df = 2, *P* = 0.60). Most fruit ripening occurred from July to December in Kona and June to December in Ka’u, with the peak harvest months for both districts being September–November (Fig. 3C). Peak harvest for low- and mid-elevation farms was in October, while peak harvest was in November for high-elevation farms (Fig. 3D).

### Coffee Berry Borer Flight Activity

**Fig. 4.**
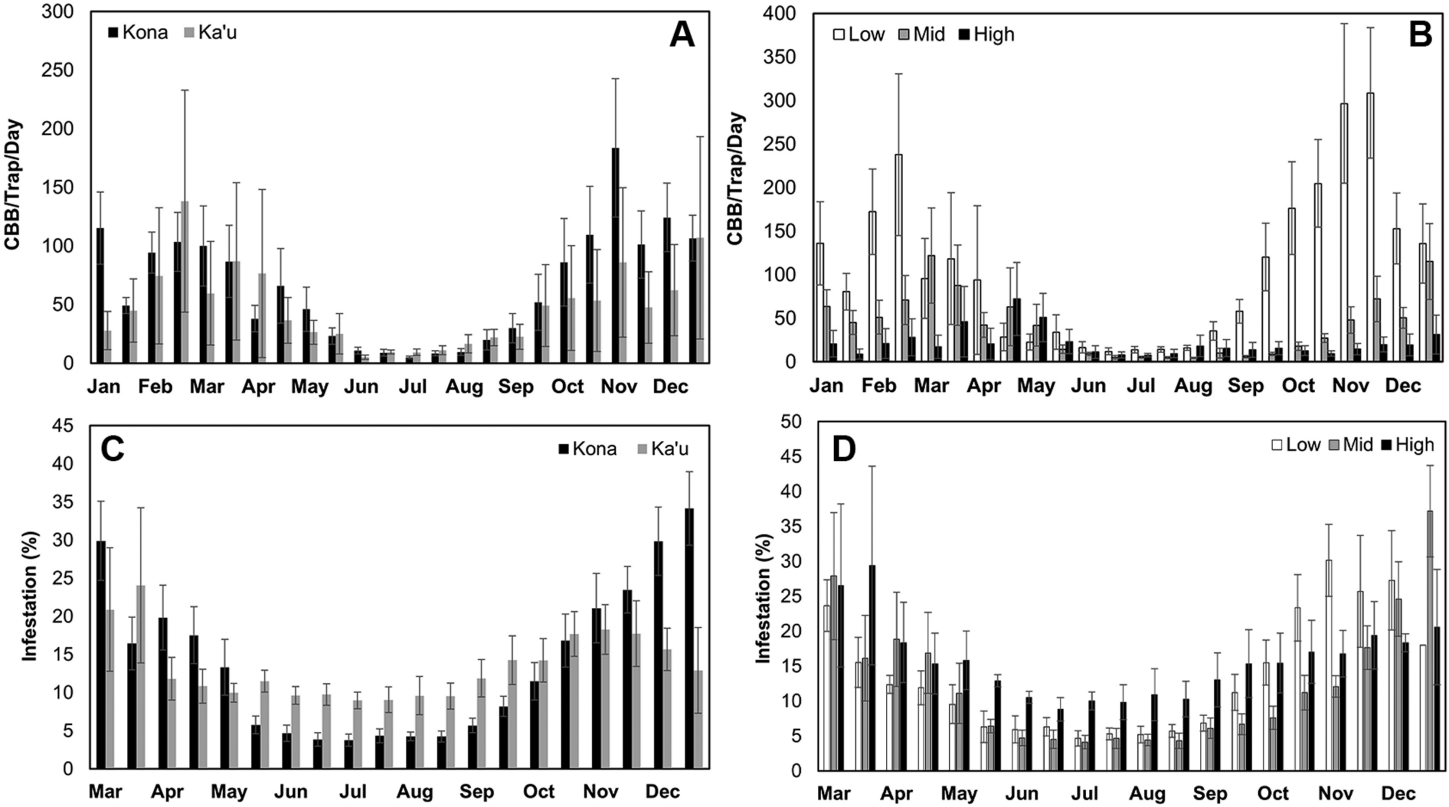
Flight and infestation dynamics of coffee berry borer on Hawaii Island; averages are shown across 2016–2018: A) trap catch in the Kona and Ka’u districts, B) trap catch at low, mid, and high elevations, C) fruit infestation in the Kona and Ka’u districts, and D) fruit infestation at low, mid, and high elevations. Error bars represent ± 1 standard error.

The average trap catch in Kona was significantly higher than in Ka’u (65.74 vs. 48.05 coffee berry borer/trap/day; *V* = 225, *P* = 0.03) (Fig. 4A). The mean difference in trap catch was more than twice as high in the second half of the season (fruit maturation and harvest) compared to the first half of the season (postharvest and fruit development). The average trap catch was also significantly different among elevations (χ^2^ = 17.42, df = 2, *P* < 0.001) (Fig. 4B). A Tukey multiple comparisons test showed that low-elevation farms captured significantly higher numbers of coffee berry borer (107.56 coffee berry borer/trap/day) compared to mid- (41.16 coffee berry borer/trap/day; *P* < 0.001) and high-elevation farms (21.49 coffee berry borer/trap/day; *P* < 0.001).

### Infestation and Coffee Berry Borer Position

**Fig. 5.**
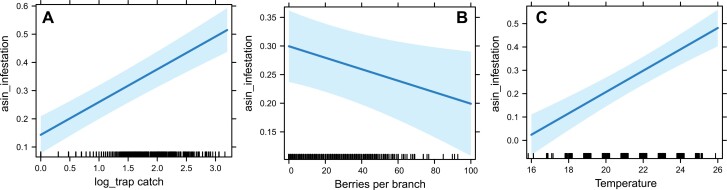
The effect of A) coffee berry borer trap catch, B) fruit availability, and C) air temperature on coffee fruit infestation. Fitted parameter estimates are shown with a 95% confidence interval (shaded). The distribution of data points is shown with tick marks at the bottom of each graph.

Mean coffee fruit infestation was not significantly different between Kona and Ka’u (13.94% vs. 13.42%; *V* = 106, *P* = 0.98) (Fig. 4C). For both districts, infestation was highest in March–April and September–December, corresponding to early fruit development and harvest, respectively. Fruit infestation also did not differ among elevations (low = 13.53%, mid = 12.36%, high = 15.74%; χ^2^ = 4.13, df = 2, *P* = 0.13) (Fig. 4D). According to a reduced GLMM (log-likelihood = 181.83, full model log-likelihood = 169.38), coffee berry borer pressure (i.e., trap catch) and air temperature had a significant positive effect on infestation, while fruit availability had a significant negative effect on infestation (Fig. 5; Table 2). Conversely, rainfall and RH had no effect on infestation (*P* > 0.05; data not shown).

**Table 2.** Generalized linear mixed model estimates for the effect of trap catch (log-transformed), fruit availability, and air temperature on coffee berry borer infestation. Trap catch and air temperature had a significant positive effect on infestation, while fruit availability had a significant negative effect on infestation

	Value	SE	*t*-value	*P*-value
Trap catch	0.116	0.012	9.342	<0.001
Fruit availability	–0.001	0.000	–2.242	0.0252
Temperature	0.046	0.006	7.881	<0.001

**Fig. 6.**
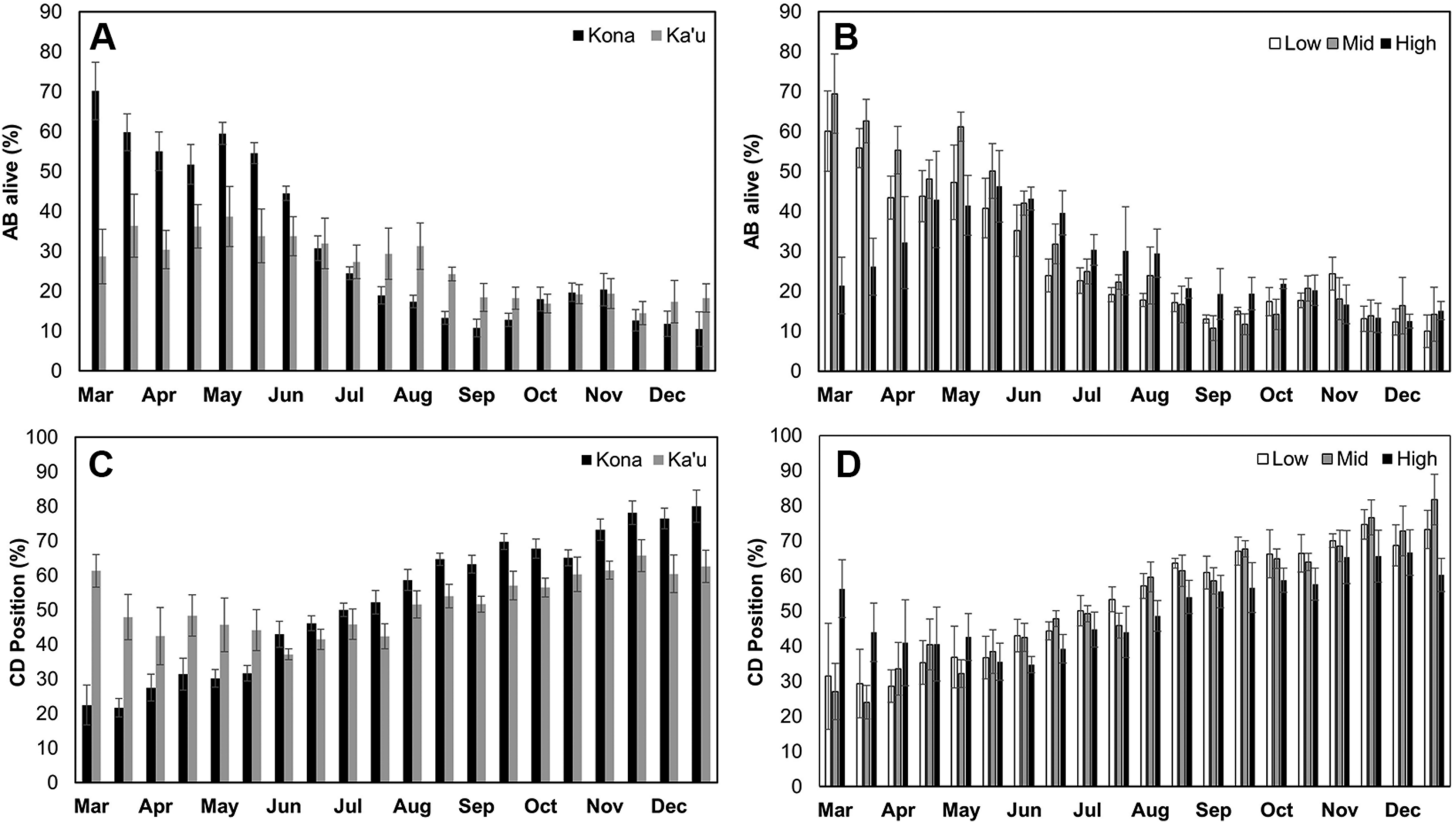
Position of coffee berry borer within the fruit; averages are shown from 2016 to 2018 on Hawaii Island: A) alive and in the AB position (early boring stage, prior to entry of the endosperm) in the Kona and Ka’u districts, B) alive and in the AB position at low, mid, and high elevations, C) in the CD position (bean damage) in the Kona and Ka’u districts, and D) in the CD position at low, mid, and high elevations. Error bars represent ± 1 standard error.

The percentage of coffee berry borer that were alive and in the AB position was not different between districts (Kona = 30.84% vs. Ka’u = 26.23%; *V* = 123, *P* = 0.52; Fig. 6A) or among elevations (low = 27.51%, mid = 31.43%, high = 27.12%; χ^2^ = 0.34, df = 2, *P* = 0.85; Fig. 6B). However, the peak in AB alive was in March for Kona farms and low and mid-elevations, compared to May for Ka’u and high elevations. The percentage of coffee berry borer that were in the CD position was also not different between districts (Kona = 52.67 vs. Ka’u = 51.92%; *V* = 113, *P* = 0.78; Fig. 6C) or among elevations (low = 52.86%, mid = 52.87%, high = 50.61%; χ^2^ = 0.50, df = 2, *P* = 0.78; Fig. 6D). Coffee berry borer damage increased as the season progressed with the peak occurring in November–December for all districts/elevations.

### Weather Variables

**Fig. 7.**
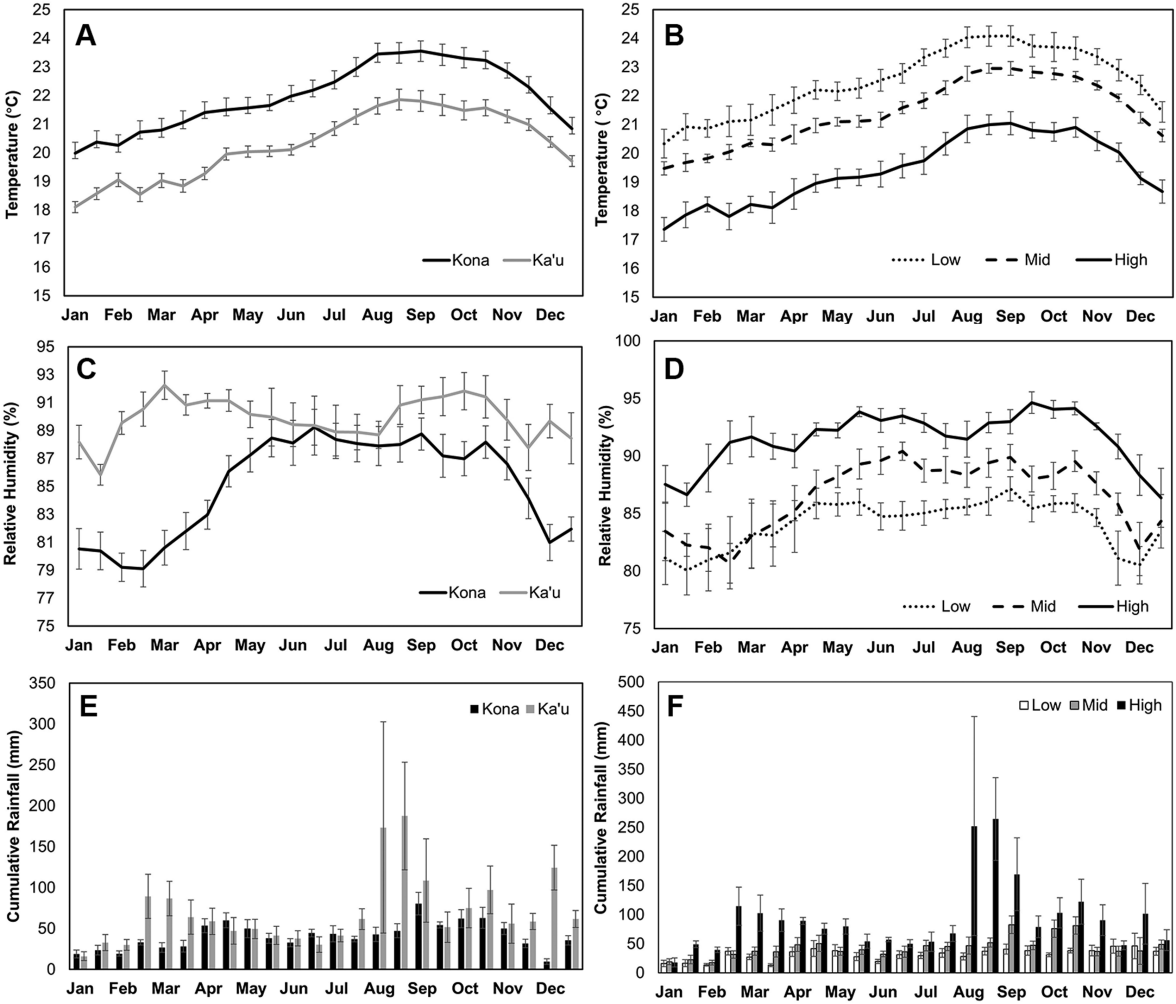
Mean values for weather variables from 2016 to 2018 on Hawaii Island coffee farms: A) air temperature in the Kona and Ka’u districts, B) air temperature at low, mid, and high elevations, C) RH in the Kona and Ka’u districts, D) RH at low, mid, and high elevations, E) cumulative rainfall in the Kona and Ka’u districts, and F) cumulative rainfall at low, mid, and high elevations. Error bars represent ± 1 standard error.

Across the entire season, Kona was on average 1.68 °C warmer than Ka’u (21.95 °C vs. 20.27 °C), and this difference was statistically significant (*V* = 300, *P* < 0.001) (Fig. 7A). Peak temperatures occurred from August to October in both districts. There was also a significant difference in mean air temperature among all 3 elevations (low = 22.50 °C, mid = 21.39 °C, high = 19.41 °C; χ^2^ = 40.45, df = 2, *P* < 0.001) (Fig. 7B). RH was significantly different between districts (*V* = 0, *P* < 0.001; Fig. 7C), with the average RH being 4.85% higher in Ka’u compared to Kona (89.88% vs. 85.03%). The largest difference in RH between the 2 districts was observed early (January–March) and late in the season (September–December), while averages were similar during the summer months (June–August). A significant difference in mean RH was observed among all 3 elevations (low = 84.09%, mid = 86.51%, high = 91.46%; χ^2^ = 43.22, df = 2, *P* < 0.001) (Fig. 7D). However, the peak in RH was from April to October in all 3 elevations. The mean biweekly rainfall total was higher in Ka’u compared to Kona (70.06 vs. 41.06 mm; *V* = 34, *P* < 0.001) (Fig. 7E). Peak rainfall was recorded in August for Ka’u and September for Kona, while the lowest rainfall was recorded in January for both Kona and Ka’u. Rainfall differed significantly among elevations (χ^2^ = 37.22, df = 2, *P* < 0.001), with low- and mid-elevation farms having lower biweekly averages relative to high-elevation farms (*P* < 0.001; Fig. 7F).

### Management Practices

On Kona farms, pruning was often conducted in January (range = January–March), while pruning on Ka’u farms occurred on average in April (range = January–July). The majority of farms (12 of 14) used the traditional Kona style of pruning (multiple verticals of different ages on each tree, with 1–2 of the oldest verticals removed each year), while only one used the Beaumont–Fukunaga style (multiple verticals of the same age, with every 2–3 rows stumped in a 3- to 5-year cycle), and one farm used the umbrella style of pruning (trees are topped annually to promote lateral growth) (see Bittenbender and Smith 2008). Kona farms sprayed for coffee berry borer 6.05 ± 0.62 times per year on average compared to 4.13 ± 1.13 times per year in Ka’u. On average, Kona farms began spraying for coffee berry borer in March, while Ka’u farms began spraying in April; the last spray of the season was typically conducted in September for farms in both districts. Low elevation farms sprayed for coffee berry borer 7.11 ± 0.92 times per year on average, compared to 5.70 ± 0.88 times at mid-elevation farms, and 3.43 ± 0.68 times at high-elevation farms. The mean number of harvesting rounds (including sanitation picks, standard harvesting, and strip-picks) was higher in Ka’u (9.63 ± 1.68 rounds) compared to Kona (6.84 ± 0.75 rounds). However, the number of harvesting rounds did not differ among elevations, with the average being 8 rounds. Strip-picks were typically conducted in December (range = December–January) for Kona farms, compared to March (range = December–May) on Ka’u farms.

### Spray Determination

Based on the data collected across 14 farms on Hawaii Island, our estimates for spray determination were highly variable, although there were more similarities within elevation categories than within districts (Table 3). For Kona, we estimate that 3–7 sprays are optimal for low-elevation farms, 4–5 sprays for mid-elevation farms, and 2–3 sprays for high-elevation farms (Table 3). For Ka’u, we estimate that 2–8 sprays are optimal for low-elevation farms, 3–6 sprays for mid-elevation farms, and 0–4 sprays for high-elevation farms (Table 3). The optimal spray window for controlling coffee berry borer in Ka’u is slightly longer relative to Kona, reflecting the year-round season. In general, the number of sprays needed to control coffee berry borer decreases with increasing elevation.

**Table 3.** Results of the spray determination scoring matrix based on a combination of trap catch, fruit production, infestation, and coffee berry borer in the AB position. Location includes district (Kona or Ka’u) and elevation (low, mid, or high) for Hawaii Island coffee-growing regions. Scores were out of a possible 24 points and ranked according to spray priority for each month: critical (C = 20–21), high (H = 18–19), medium (M = 16–17), or low (L < 16). January and February were not included in the spray calendar since plants are typically either dormant or flowering at this time

Location	March	April	May	June	July	August	September	October	November	December
Kona Low	M	M	C	H	M	M	H	L	L	L
Kona Mid	M	H	C	H	L	L	L	H	L	L
Kona High	L	M	H	C	L	L	L	L	L	L
Ka’u Low	M	H	H	L	M	M	M	M	M	L
Ka’u Mid	M	H	M	H	M	C	L	L	L	L
Ka’u High	L	L	L	M	M	M	M	L	L	L

## Discussion

In the present study, we measured significant differences in coffee berry borer population dynamics, plant phenology, and weather across the heterogeneous coffee-growing landscape on Hawaii Island. These suggest improvements to the timing of pesticide sprays to control coffee berry borer, thereby increasing the efficiency of control and cost-effectiveness of coffee production on Hawaii Island. Instead of a one-strategy-fits-all IPM for coffee berry borer, our results strongly indicate that management should be tailored to the conditions of the specific growing location. Making spray decisions based on measurements of the local weather and plant phenology on the farm will be the most successful strategy for managing this global pest. However, even without measurements from a given farm using patterns particular to a given region and elevation will be advantageous. The implementation of a coordinated area-wide approach refined by location- or farm-specific optimized IPM will lead not only to improved management of coffee berry borer on individual farms, but a reduction in pest pressure across the coffee-growing landscape.

We observed significantly larger populations of coffee berry borer in Kona compared to Ka’u over the 3 study years based on biweekly trap catches. Larger coffee berry borer populations were also observed at low elevations compared to mid and high elevations. These data are in line with the study by Hamilton et al. (2019), which reported faster coffee berry borer development times in Kona compared to Ka’u due to a narrower range in mean daily temperatures in the Kona district. Those authors also reported that coffee berry borer development was faster at low elevations in Hawaii, with an estimated 4–5 coffee berry borer generations per season at low elevations (<400 m) compared to 2–3 generations per season at high elevations (>600 m). In contrast to our findings, Aristizabal et al. (2017b) reported higher numbers of coffee berry borer from traps in Ka’u vs. Kona. However, this result is likely due to several factors including a single season of data collection, the inclusion of only mid-elevation farms in Ka’u, differences in management practices among farms, and random differences among the farms and years sampled.

Although larger populations of coffee berry borer were observed in Kona and at low-elevation farms, we found no significant difference in infestation between districts or elevations. The fact that infestation was not significantly higher in Kona and at low elevations despite higher pest pressure and temperatures may be because these locations had higher fruit production, which would offset an increase in infestation. In addition, the drier conditions at low elevations and in the Kona district may reduce infestation by causing increased mortality due to the sensitivity of coffee berry borer to low humidity (Baker et al. 1994). It is important to note that RH was lowest (<80%) at these locations during periods of high flight activity (January–March, November–December), such that large numbers of coffee berry borer would have been exposed to less-than-ideal weather conditions when searching for berries and may have contributed to high mortality.

Our GLMM shows that increases in pest pressure (trap catch) and air temperature significantly increased infestation. The positive influence of air temperature on infestation is likely tied to faster rates of coffee berry borer development under warmer temperatures, and thus more generations of coffee berry borer per season (i.e., higher pest pressure; see above paragraph). In contrast, as fruit availability increased infestation significantly decreased. Coffee berry borer infestation was highest early in the season during fruit development and late in the season during harvest and postharvest. At these points in the season, fewer fruits were available to the large numbers of recently emerged coffee berry borer that were actively flying and searching for berries, resulting in high infestation percentages.

Variations in cultural management practices that are essential for the effective control of coffee berry borer were evident across farms. The traditional Kona style of pruning was used at 12 of 14 farms, with one grower each implementing Beaumont–Fukunaga or umbrella pruning. Importantly, these 3 styles of pruning do not effectively reduce the coffee berry borer population since productive branches remain between seasons. Stump pruning by block is the only pruning method that can eliminate the supply of food and shelter for coffee berry borer by removing all branches from the trees in the entire block (Aristizábal et al. 2017a, Kawabata et al. 2020). However, not all farmers can afford to lose production for 1–2 seasons, and/or may have older trees or drought conditions that could prevent stump pruning. We observed stump pruning in one study farm in Kona and 3 farms in Ka’u after the completion of study, suggesting that some growers are starting to utilize this method when feasible. In general, stump pruning by block will be more economically feasible for large farms (>5 acres) with multiple blocks such as those in Ka’u, to allow for at least some production while the stumped blocks regenerate.

We also observed more frequent harvesting in Ka’u relative to Kona, which reflects the year-round production in this district. Frequent and efficient harvesting has been shown to be effective at limiting coffee berry borer infestation and bean damage compared to the conventional strategy of frequent pesticide sprays and few harvesting rounds (Aristizábal et al. 2023b). In contrast, strip-picking at the end of the season was more frequently done in Kona compared to Ka’u, again due to the nature of the year-round season in Ka’u. Growers in Ka’u are less willing to strip-pick trees because there is no clear end to the season, meaning that some developing berries would be removed, and revenue lost. For this district, frequent sanitation picks of ripe, over-ripe, and raisin berries throughout the season could be a viable alternative to strip-picking (Aristizábal et al. 2023b). To optimize coffee berry borer control, these cultural practices of pruning, harvesting, and sanitation must be carefully considered and combined with properly timed pesticide sprays (Johnson et al. 2019, Wraight et al. 2021, Aristizábal et al. 2023b).

When considering the combined information on coffee berry borer flight activity, fruit phenology and production, infestation, and coffee berry borer position within the fruit, a spray schedule that is optimized for each location will likely lead to improvements in efficiency. Low-elevation farms could time their sprays to occur both in the early season to protect the developing fruit, as well as later in the season to reduce damage resulting from subsequent generations emerging during the harvest. With fewer generations of coffee berry borer expected at elevations > 400 m, mid-elevation farms could focus on spraying in the early and mid-season from March to August. Lastly, high-elevation farms could time sprays to begin slightly later than those at low- and mid-elevations, starting in April (Kona) or June (Ka’u) and continuing for a few months.

Two previous studies conducted in Hawaii have suggested strategies to optimize sprays for coffee berry borer control. Hollingsworth et al. (2020) reported that 4–5 sprays done earlier in the season based on an action threshold were just as effective as 7–11 sprays conducted throughout the year based on a calendar strategy. Woodill et al. (2021) examined 4 strategies for spraying: never spray, always spray, IPM choice, and economic model. The IPM choice strategy was based on the most recent University of Hawaii extension service IPM recommendations for coffee berry borer in Hawaii, which only considers the percent infestation and AB alive for spray determination (see Table 1 in Kawabata et al. 2020). Woodill et al. (2021) reported that following the IPM choice strategy resulted in spraying 10 months out of the year (January–October) when using data on infestation and AB and CD position. In contrast, the economic model resulted in 6 sprays (June-November) and provided a higher net benefit in comparison to the other 3 strategies. The IPM choice performed only slightly better than always spraying, and both were better than never spraying. The authors suggested that a hybrid combination between the IPM recommendations and calendar spray approaches could improve farm-level decisions (Woodill et al. 2021).

Applying the IPM strategy mentioned above based on the data collected in both districts for the present study results in low- and high-elevation farms spraying 12 months out of the year and mid-elevation farms spraying 9 months out of the year. Using additional information on coffee berry borer flight and coffee plant phenology, we find that conservatively sprays could be reduced by 33–42% for low-elevation farms, 33–44% for mid-elevation farms, and 67–75% for high-elevation farms. In general, growers at low elevations and in regions with a year-round growing season will need to conduct more frequent sprays relative to growers at mid- and high-elevations and in regions with a distinct dry season.

While the data collected for this study allow us to propose spray schedules optimized by region and elevation, decisions for any coffee farm can be improved further by monitoring conditions on the farm as well as the population of coffee berry borer. This can be achieved by collecting weather and trapping data, quantifying phenology, incorporating natural enemy effects, and tracking infestation rates and position of coffee berry borer. Growers utilizing data-driven decision support tools might be able to target their ideal spray times even more precisely than the improved recommendations here, increasing the economic return of the operations.

Although the importance of early-season sprays to protect the young developing fruit has been discussed in several articles (Aristizábal et al. 2016, 2023b, Hollingsworth et al. 2020, Kawabata et al. 2020, Wraight et al. 2021), our study is the first to show that 1–2 sprays conducted during the main harvest would be beneficial for low-elevation farms and those farms with a year-round season, particularly when fruit loads are high. This is also the first study to our knowledge that provides growers with a spray calendar that can be used to guide management strategies depending on the farm location. Although slight variation may occur in flight peaks and phenology from year to year, this location-specific guide may be used to give growers a general idea of the best months to target coffee berry borer. The information presented here provides growers with a better understanding of when sprays should occur during the season to maximize benefits based on peak coffee berry borer flight times (i.e., exposure to pesticides) and availability of fruit. With the continued threat of new pests and diseases, an ever-changing climate, and rapidly rising production costs, optimizing the timing and number of sprays is more important than ever for coffee growers worldwide.

## Supplementary Material

toae061_suppl_Supplementary_Information_S1

## Data Availability

Data from this study are available from the USDA Ag Data Commons repository: https://doi.org/10.15482/USDA.ADC/25347559.v1 (Johnson and Manoukis 2024).
